# On Our Knowledge of Thiol

**Published:** 1889-10

**Authors:** F. Buzzi

**Affiliations:** Assistant to Prof. Schweninger in the University Clinic in Berlin


### ON OUR KNOWLEDGE OF THIOL.

By DR. F. BUZZI,

Assistant to Prof. Schweninger in the University Clinic in Berlin.

*Translated from Monatshefte fur praktisehe Dermatologie.*

In a treatise on "Thiol and Ichthyol" (Mitth. a. d. dermatol. Clin. der Charite in Berlin.) Dr. L. Reeps called attention to the physical, chemical and therapeutical identity of these two preparations. Reeps claim for Thiol, recently discovered by Dr. Emil Jacobsen, the same therapeutic action and value as possessed by the well-known and successfully introduced Ichthyol. The practical experiences on which he bases this claim were limited to a few cases only, but this was due primarily to inability to procure Thiol, which up to date had been produced only in limited quantities in a private laboratory.

Of late, however we have resumed these researches in our clinic, polyclinic, and private practice, as ample supplies are now obtainable, manufactured in large quantities for general consumption. It is true that my experments are not yet completed either, and, I must postpone publication of a final report until some future occasion; but I have no hesitancy in endorsing all that Reeps claimed for the efficacy of Thiol in those diseases which I have treated with it: seborrhœa, rosacea, acne, eczema etc. Thiol fully possesses all the widely and authoritatively claimed therapeutic virtues of Ichthyol, especially as indicated in Unna's excellent treatise: Ichthyol and resorcin as representatives of the class of reducing remedies (*Dermatol. Studien*, Heft 2, 1889)—as is really self-evident, from the fact that both remedies are chemically identical. The chief properties of Thiol, therefore are its absorbent, styptic and antiseptic action. With reference to above experiments by Reeps I will give my experience in characterizing Thiol, which I was able to conduct with the assistance of Dr. Jacobsen; the article being readily procurable in the market, its manufacturer having overcome numerous difficulties, I was also able to get it in a chemically pure state. Therefore, considering the source from which Reeps obtained his solution of thiol, and its comparative impurity, we must certainly make some allowance. Whether an ideal thiol should be composed of one unsaturated carbo-hydrate, and if such were preferable to a mixture of thiols, I do not here wish to discuss; but am satisfied to know that thiol, as well as ichthyol is a mixture of carbo-hydrates which is turned into a soluble sulphate (in water) by treatment with sulphuric acid. Ideal thiol would be of only theoretical value, as in the first place, no one, to the best of my knowledge, has yet been able to isolate an unsaturated carbo-hydrate; secondly, it can safely be assumed that the carbo-hydrates of iodol as well as ichtyol do not affect the remedy therapeutically. The main point lies in their combination with sulphur (making it soluble in water), and as it requires a percentage amount of sulphur to enter into the combination of the carbo-hydrates, we do not suppose the preparations would differ much, thereapeutically speaking (thiol or ichthyol). Should, however, these investigations of carbo-hydrates be followed more closely, we would eventually arrive at an ideal thiol, which would outshine its predecessors and would lead to as many thiols as we have unsaturated carbo-hydrates. The possibility of this I will readily admit, at the same time would say that thiol of the present time is fast pushing the road to an ideal thiol, for which we must give credit to the indefatigable Jacobsen, who has shown us that in the preparation of this article the sulphureting of crude oil is merely a special step accompanying the ordinary reaction. What are our conclusions of the above in a practical way? Using ichthyol, we find the mixture of sulphureted carbo-hydrates effective, and no one has asserted as yet that this is due to any single body it contains. The same we say of thiol, and therefore their identity, as both represent virtually a mixture of unsaturated sulphureted carbo-hydrates. The same idea we often meet in combinations of medicines, whose combinations result in the efficiency, as in solid extracts, opium, cod liver oil, and the newly discovered kreolin. As it is of most importance to have a uniform preparation (for its therapeutical use) Jacobsen uses a raw material, which by experience has proven itself the best, and places its specific gravity and boiling point at close range.

During the preparation of thiol we meet a number of foreign bodies, which show acid reactions, but yet can only be looked upon as impurities. The present article is now free of these impurities, which originate in the process of acidulation, also being freed from the crude oil by the use of ligroin. Above all, thiol does not possess the objectionable odor of crude oil, which most preparations retain tenaciously. The odor of C. P. thiol is slightly bituminous, not in the least offensive, the taste being slightly astringent and the reaction on litimus paper neutral. It is unnecessary for me to go into further details, but I would merely state that thiol is now procurable in two forms, viz.: 1. Thiol liquidum, a 40 per cent aqueous solution, the consistency of a thick syrup of sp. gr. 1080 to 1081 ($15_0$ C). This preparation, owing to its similarity to ichthyol, will probably be dispensed.

2. Thiolium siccum. A carefully dried thiol in the lamellated shape, dark brown in color and shiny. For convenience in dispensing, it is put into a subtillated form. The possibility of having a solidified (dry). Thiol has only been attained by removing all impurities and contaminating salts, which avoids all hygroscopic tendencies, and results in the permanent solidified form. This preparation is soluble in water without decomposing. The astringent taste of thiol is especially noticeable in this form. Not alone that in this form we have a preparation which is entirely free of water, a matter sometimes of the utmost importance, we can hail it as a remedy for internal use, and therefore far superior to ichthyol. Beside, it can also be used as a dusting powder in affections of the skin, where absorbents are of special value, principally those which are introductory to eczema (as eryth. caloric., eryth. ex profluviis), also for erysipilas, fresh burns, blisters, as in pemphigus, darmatitis herpetiformis, impetigo, etc. For this purpose a 10 to 20 per cent mixture is used, being mixed with either talcum, starch, or oxide of zinc, as occasion may require. Considering the favorable experience of unna with ichthyol as a healing agent for wounds of the skin, it should be but a short time before thiol will be used in the practice of surgery, as Kocher recommends bismuth. subnitr. Its use as a dusting powder would naturally be regulated by its cost. This very thing induced me to make researches, and I consider it a most important item. We were often compelled in our polyclinics to dispense with the use of ichthyol owing to its high price. As we now have two preparations of equal value, it will naturally produce competition, and thereby reduce prices. This end may be easily attained if some of our more prominent members of the profession, not only dermatologists but regular practitioners, will convince themselves by using it, of its comparative efficiency with ichththyol.—*Notes on New Remedies.*

